# What Are the Reasons for Continuing Failures in Cancer Therapy? Are Misleading/Inappropriate Preclinical Assays to Be Blamed? Might Some Modern Therapies Cause More Harm than Benefit?

**DOI:** 10.3390/ijms232113217

**Published:** 2022-10-30

**Authors:** Razmik Mirzayans, David Murray

**Affiliations:** Department of Oncology, University of Alberta, Cross Cancer Institute, Edmonton, AB T6G 1Z2, Canada

**Keywords:** cancer therapy, intratumor heterogeneity, polyploid giant cancer cells, senescence, apoptosis, anastasis, immune system, preclinical assays, multiwell plate viability assays

## Abstract

Over 50 years of cancer research has resulted in the generation of massive amounts of information, but relatively little progress has been made in the treatment of patients with solid tumors, except for extending their survival for a few months at best. Here, we will briefly discuss some of the reasons for this failure, focusing on the limitations and sometimes misunderstanding of the clinical relevance of preclinical assays that are widely used to identify novel anticancer drugs and treatment strategies (e.g., “synthetic lethality”). These include colony formation, apoptosis (e.g., caspase-3 activation), immunoblotting, and high-content multiwell plate cell-based assays, as well as tumor growth studies in animal models. A major limitation is that such assays are rarely designed to recapitulate the tumor repopulating properties associated with therapy-induced cancer cell dormancy (durable proliferation arrest) reflecting, for example, premature senescence, polyploidy and/or multinucleation. Furthermore, pro-survival properties of apoptotic cancer cells through phoenix rising, failed apoptosis, and/or anastasis (return from the brink of death), as well as cancer immunoediting and the impact of therapeutic agents on interactions between cancer and immune cells are often overlooked in preclinical studies. A brief review of the history of cancer research makes one wonder if modern strategies for treating patients with solid tumors may sometimes cause more harm than benefit.

## 1. Introduction: Why Is the ‘War on Cancer’ Not Won Yet?

An editorial article was recently published in Nature that provides an update on the progress made in cancer research since the Congress of the United States passed the National Cancer Act in December of 1971 [1]. The article is entitled “The ‘war on cancer’ isn’t yet won” and highlights how the National Cancer Act has fostered tremendous advances, in large part through a greater understanding of the mind-boggling complexity of the biology that underlies the disease, as well as methodology development for cancer research that has spilled over into other fields, such as the discovery of ways to treat COVID-19. The article also underscores the fact that after half a century of extensive research and numerous clinical trials, cancer remains the second-leading cause of death in the United States and, by inference, in other developed countries.

This shocking reality check raises a fundamental question: What lessons have we learned from this past half century of recurrent failures in cancer therapy, including recent chemo-immunotherapy of solid tumors [2], that we need to take into consideration to avoid repeating the same mistakes and expecting different results in the years to come? We decided to write the current brief review to consider this question.

The current article covers the following four topics: (i) well established and yet widely unappreciated responses that contribute to therapy resistance (Section 2); (ii) a brief history of cancer chemotherapy since the early 1970s, as outlined by Robert Weinberg in 2014 [3], and more recent discoveries that have shed light on the reasons why preclinical anticancer studies often generate clinically irrelevant information (Section 3); (iii) dishonesty in cancer research (Section 4); and (iv) the impact of modern cancer therapies, with and without surgery, on the survival of patients with solid tumors and the impact of the host immune system thereon (Section 5, Section 6 and Section 7).

One of our objectives is to present sufficient evidence to appreciate the danger of relying on rather simplistic, short-term and frequently misleading preclinical anticancer activity assays which have been employed by many cancer researchers, including our own group prior to approximately a decade ago (reviewed in [4]), to win the metaphoric “war on cancer”.

## 2. Well Established and Yet Widely Overlooked Therapy-Induced Cancer Cell Responses That Contribute to Disease Recurrence

### 2.1. Cell Recovery from Death through Anastasis

Over two decades ago, Geske and colleagues reported on a series of experiments demonstrating that p53-dependent apoptosis can be reversible [5,6]. The authors used a mouse mammary cell line in which wild-type p53 function was temperature sensitive. Activation of p53 promoted apoptosis, as indicated by phosphatidylserine externalization and DNA fragmentation, based on flow cytometric (Annexin V staining) and TUNEL (terminal deoxynucleotidyl transferase-mediated dUTP nick end labeling) assays, respectively [5,6]. These cultures were shown to maintain their proliferative capacity when they were subsequently incubated under conditions to remove the apoptotic stimulus (i.e., p53 activation), suggesting that early stages of p53-induced apoptosis were reversible in this mouse cell-line model.

The observation of Geske et al. [5,6] that triggering apoptosis might not always lead to cell demise went largely unnoticed except for occasional mentions in review articles (e.g., [7]). Since 2009, however, numerous groups have independently reported the phenomenon of recovery from death in various biological systems (reviewed in, e.g., [4,8]). These include human solid tumor-derived cell lines with differing p53 status after treatment with chemotherapeutic drugs under conditions that were comparable to how drugs are administered to patients [9,10,11,12,13,14,15,16,17]. Namely, they involved short duration of exposure to moderate concentrations of chemotherapeutic drugs followed by incubation in drug-free medium (for details regarding clinically relevant chemotherapy exposure, please see [18,19]).

The process of mitochondrial outer membrane permeabilization (MOMP) has been assumed to represent the point-of-no-return for many cell types triggered to die through apoptosis [20,21]. It has therefore been proposed that perhaps recovery from death might occur at early stages of apoptosis and only in cells exhibiting “limited” MOMP (i.e., when only a fraction of the mitochondria in a cell were permeabilized and the majority remained intact). In 2019, Seervi and associates tested this possibility in solid tumor-derived cell lines following treatment with relatively high concentrations of the chemotherapeutic drugs paclitaxel (200 nM, 24 h) and etoposide (100 μM, 24 h) [13]. Recovery was observed not only after “limited” MOMP, but also after “widespread” MOMP (i.e., when most of the mitochondria in a cell were permeabilized). Although only a small proportion (2–5%) of cancer cells with widespread MOMP were shown to recover under the experimental conditions used by these authors, such cells exhibited enhanced motility and invasiveness.

Collectively, these observations demonstrate technical challenges in assessment of cancer cell death through apoptosis. They also suggest that the previous reports that have relied on apoptosis-associated events (e.g., MOMP, caspase activation, phosphatidylserine externalization, DNA fragmentation) as markers of cancer cell death need to be reassessed to distinguish dead cancer cells and dying cancer cells that can recover from the brink of death.

The phenomenon of recovery from death is now being referred to as anastasis (Greek for “rising to life”). Anastasis appears to involve physiological healing processes, but unfortunately it facilitates cancer cell survival following therapeutic exposures. The potential challenges in the treatment of solid tumors associated with anastasis have been recently discussed [4,8,22,23,24,25,26,27,28].

*Update:* To the best of our knowledge, the work presented by Seervi and coworkers in 2019 [13] was the last reported research article on anastasis. We did PubMed searches to find research reports on this topic and noticed that the majority of recent publications (since 2019) on cancer cell response to therapeutic agents have not only overlooked anastasis but have also continued to use caspase activation, Annexin V staining, and/or positive TUNEL staining as markers of cancer cell death. Since 2019, however, there have been several comprehensive reviews on the potential dark side of anastasis in cancer therapy and how this response complicates the interpretation of results obtained by standard radiosensitivity and chemosensitivity assays in terms of cancer cell death (e.g., [4,8,25,26,27,28]).

### 2.2. Pro-Survival Functions of Caspase-3

In addition to their ability to recover from the brink of death, apoptotic cells can stimulate compensatory proliferation via various mechanisms [29,30,31,32,33,34,35,36,37,38,39,40,41,42,43,44,45,46,47,48]. These include caspase-3-mediated secretion of pro-survival factors (e.g., prostaglandin E_2_) that promote enrichment of tumor repopulating cells [37,38,39,40,41], and caspase-3/DNase-mediated accumulation of genome instability (e.g., DNA double-strand breaks) that trigger activation of transcription factors such as NF-κB and STAT3 that drive tumor growth [42,43]. These proliferation-stimulating pathways have been referred to as “Phoenix Rising” [29,37,43,45] and “Failed Apoptosis” [46,47,48]. While these pathways, like anastasis, represent important recovery processes in normal tissue, they can also nullify pro-apoptotic therapy of solid tumors. In short, instead of functioning as a death executioner in the apoptotic pathway, active caspase-3 can promote carcinogenesis, metastasis, and therapy resistance.

*Update:* Although multiple functions of caspase-3 have long been established, including its pro-survival and tumor promoting properties, immunoblot detection of active caspase-3 continues to be used by many authors as a marker of cancer cell death without validation by other biomarkers to distinguish dying and dead cells.

### 2.3. Impact of Therapy-Induced Cancer Cell Dormancy through Polyploidy, Multinucleation and/or Premature Senescence on Disease Recurrence

As highlighted by Herbein and Nehm [49], “Tumors are renowned as intricate systems that harbor heterogeneous cancer cells with distinctly diverse molecular signatures, sizes and genomic contents. Among those various genomic clonal populations within the complex tumoral architecture are the polyploid giant cancer cells… (that) are increasingly being recognized for their prominent role in tumorigenesis, metastasis, therapy resistance and tumor repopulation after therapy.”

Although described over a century ago, it was not until the late 1950’s when Puck and Marcus reported that giant cells that developed in cultures of the HeLa cervical carcinoma cell line, following exposure to ionizing radiation, remain viable and secrete growth promoting factors (reviewed in [50]). Since then, the basis for the creation of giant cancer cells with massive nuclear contents and their impact on therapy resistance and disease relapse has been clearly established and extensively reviewed (e.g., [49,50,51,52,53,54,55,56,57,58,59,60,61,62,63]).

Giant cancer cells with a highly enlarged nucleus, multiple nuclei (sometimes more than ten), and/or multiple micronuclei have been variously referred to as Multinucleated Giant Cancer Cells (MNGCs), Giant Aneuploid Cancer Cells (GACCs), and Polyploid Giant Cancer Cells (PGCCs). Here, we will use the term “PGCCs” for simplicity. Some images of giant cells from our published work are reproduced in Appendix A.

Below we will outline some important observations with PGCCs that are accepted by the scientific community. For further details please consult our recent reviews on PGCCs [4,50] and additional references as indicated.

**Figure 1:**
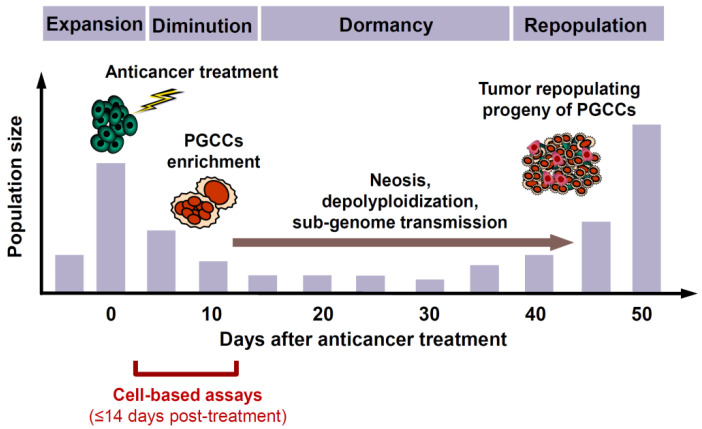
Cartoon illustrating the role of polyploid/multinucleated giant cancer cells (PGCCs) in tumor response to anticancer agents. Although anticancer treatment results in initial tumor shrinkage and a dormant state several weeks post-treatment (e.g., ~35 days in a rat colon carcinoma model [67]), it also triggers the creation of PGCCs that give rise to therapy resistant and tumor repopulating progeny through neosis (nuclear budding and bursting), depolyploidization involving meiosis and self-renewal genes, and sub-genome transmission (transfer of nuclear material into surrounding cells via cytoplasmic tunnels). For further details, please consult [50].

- PGCCs are present in solid tumors/tumor-derived cell lines with differing p53 status, and their frequency typically increases under hypoxia or following treatment with anticancer agents [50]. Non-genotoxic agents are also known to promote the creation of PGCCs. These include nutlin-3a [64], a small molecule activator of wild-type p53, and staurosporine [65], which is commonly used as a standard apoptosis-inducing agent.
- A subset of PGCCs exhibit senescence-like features, including positive staining in the senescence-associated β-galactosidase (SA β-Gal) assay [50,66,67,68,69]. SA β-Gal-positive and -negative PGCCs can be present in the same culture of a cancer cell line [50] (also see Appendix B).
- PGCCs can be created through endoreduplication (replication of chromosomes without subsequent cell division) and cell fusion [4]. Fusions between cancer cells and cancer cells, cancer cells and leukocytes, cancer cells and stem cells, and cancer cells and stromal cells are all known to promote tumor progression and therapy resistance [4].
- Although PGCCs created following treatment with anticancer agents initially (within the time span of conventional preclinical assays) cease to proliferate, they remain viable and metabolically active [50].
- Subsequently, PGCCs can not only undergo depolyploidization and resume mitosis, but they are also capable of giving rise to therapy-resistant and tumor repopulating cells through processes such as neosis (nuclear budding or bursting) as well as horizontal transfer of their nuclear material that contains stem-cell markers to neighboring cells [50].
- Although PGCCs are often fully manifested within ~3 days after treatment with anticancer agents and their depolyploidization and nuclear budding processes can commence at any time thereafter, it can take several weeks or months until a stable, rapidly proliferating population of daughter cells emerges that repopulates the tumor [50]. Thus, as illustrated in Figure 1, the time required between therapeutic exposure and emergence of tumor repopulating progeny of cancer cells triggered to undergoing dormancy through polyploidy and/or multinucleation is much longer than the time span of multiwell plate cell “viability” (e.g., MTT, CellTiterGlo, etc.), colony formation, and other widely used preclinical anticancer assays for cell “killing”.

The responses presented in Figure 1 are based on the work of Puig et al. [67] which involved rat colon carcinoma cells growing in vivo as tumor isografts established in immunocompetent rats, and treatment of tumor-bearing animals with cisplatin.

Cancer cells triggered to undergoing dormancy through premature senescence share features similar to PGCCs, which include the secretion of tumor promoting factors (the so-called Senescence-Associated Secretory Phenotype or SASP) and the potential for re-entry into the cell cycle [4]. Paradoxically, there is now evidence that this re-entry (reversal of proliferation arrest) can be accelerated by ectopic expression of caspase-3 or treatment with apoptosis-triggering anticancer drugs (e.g., camptothecin and the BCL2 inhibitor ABT-737) [70]. Under some conditions, depending on cell type and the level/type of genotoxic stress, solid tumor cells undergoing premature senescence can become polyploid and/or multinucleated, entering the stemness-tumor repopulation cycle [60,66,67,68,71,72,73,74,75,76]. In the work of Puig et al. [67], for example, cisplatin treatment induced premature senescence of colon carcinoma cells which was accompanied by polyploidy and emergence of tumor repopulating progeny.

It is important to note that the demonstration of the dark side of cancer cell senescence is not new. For example, over two decades ago Chang et al. [77] reported that senescence-like proliferation arrest mediated by p21^WAF1^ (p21) was reversible in a human fibrosarcoma cell line, and that the cells that recovered the ability to proliferate exhibited abnormal mitosis and endoreduplication. The authors concluded that “genetic destabilization in cells recovering from p21-induced growth arrest may conceivably play a role in carcinogenesis and tumor progression.”

*Update:* In 2020 we reviewed the literature on the impact of PGCCs on therapy resistance and disease relapse [4]. Since then, significant progress has been made in this field, including the demonstrations that: increased cellular stiffness, therapy resistance and migratory persistence of PGCCs is driven in part by dysregulation of actin cytoskeletal organization [78]; hypoxia-induced PGCCs can promote malignancy and an immune-suppressive microenvironment [79]; autophagy plays a key role in the formation and fate of PGCCs [80,81]; and chemotherapy-induced temporary cell cycle arrest (premature senescence) in cancer cells with differing p53 status can be followed by polyploidization and reprogramming, leading to changes in the expression of genes involved in meiosis and spermatogenesis regulation, resulting in the emergence of descendants that appear to arise inside PGCCs [76]. There is also recent evidence that mutation in the *SF3B1* gene can promote formation of PGCCs in the K562 leukemia cell line [82]. In addition to numerous research articles (e.g., [76,78,79,80,81,82]), in 2022, several comprehensive reviews were published that discussed different aspects of PGCCs in tumorigenesis and therapy resistance across different cancer types (e.g., [83,84,85,86]).

*Take home message:* A cancer therapy-related study (research/review/Editorial article) that merely relies on molecular, biochemical and morphological manifestations of apoptosis as a marker of cancer cell death is incomplete and potentially misleading. Similarly, a solid tumor therapy-related study that considers anticancer agent-induced proliferation arrest (resulting in loss of colony forming ability, a response referred to as reproductive “death” in the 1990′s [87]) as an indication of potentially favorable clinical outcome, is incomplete and can be dangerously misleading.

## 3. Snapshot of the History of Cancer Research

Therapy resistance responses outlined above were discovered largely by the use of high-resolution microscopy and observations for long times (weeks) following genotoxic insult. Studies considered in this section illustrate the consequences of ignoring such responses, as a result of relying on preclinical assays which are often short-term and generate population-based data averaged over a large number of cells.

### 3.1. Strengths and Limitations of the Reductionist Approach to Cancer Biology

The term “methodological reductionism” broadly refers to the idea that biological systems are most fruitfully investigated at the lowest possible level [88]. Although this approach has been tremendously successful in guiding experimental science and in generating a wealth of information, it has also revealed the immense complexity that currently confronts modern biology [89].

In 2014 the journal Cell celebrated the powers of reductionist molecular biology and its major successes. For this occasion, Robert Weinberg published a Leading Edge Essay entitled “Coming Full Circle—From Endless Complexity to Simplicity and Back Again” [3]. In this essay, Weinberg noted that “For many, generating large data sets became an almost-addictive undertaking. If two interacting entities—proteins for example—were critical to a well established biological process, imagine what studying two thousand proteins via interactome analyses would yield! And so, we entered, almost unconsciously, into the epoch of ‘‘omics’’—studying genomes, transcriptomes, proteomes, epigenomes, kinomes, methylomes, glycomes, and matrisomes—each one of which encompasses staggering amounts of accumulated information. The relative ease of generating vast amounts of data became almost irresistible” [3]. He also noted the lack of conceptual paradigms and computational strategies for dealing with these complex “omics” datasets and their integration.

Referring to studies on signaling pathways associated with, e.g., K-RAS oncogene and p53 tumor suppressor, Weinberg wrote “In truth, the research revealed nothing more than a probabilistic trend… at the time, as is still the case 15 years later, our understanding of how most of these signal-processing circuits actually operate was fragmentary… For this reason, we side-stepped the issues of signal transduction biochemistry and focused instead on biology—on the phenotypes of cancer cells and the tumors that they formed.” This led Hanahan and Weinberg to write a Leading Edge Review [90] in which they proposed six biological traits or “hallmarks” of cancer. Weinberg subsequently noted that “Thousands of articles referred to this review in the decade after it appeared—a tribute not to its writing, but instead to the profound need of so many colleagues to find some unifying themes among the ever-growing mass of observations” [3].

Weinberg concluded the 2014 Essay as follows: “… we have come full circle, beginning in a period when vast amounts of cancer research data yielded little insight into underlying mechanisms to a period (1980–2000) when a flurry of molecular and genetic research gave hope that cancer really could be understood through simple and logical reductionist thinking, and finally to our current dilemma. Once again, we can’t really assimilate and interpret most of the data that we accumulate” [3].

Unfortunately, with few exceptions, this reductionist thinking and information-generating approach continues to dominate the field of cancer therapeutics. Many authors simply disregard the various therapy-resistance responses discussed above (Section 2), and instead try to make sense of this vast amount of cancer research data to come up with a generalized model, often by ignoring context dependency such as types/amounts of genotoxic stress, clinical relevance of the laboratory experimental set up (e.g., duration/amount of chemotherapeutic drug treatment), and the biological system under study.

**Figure 2:**
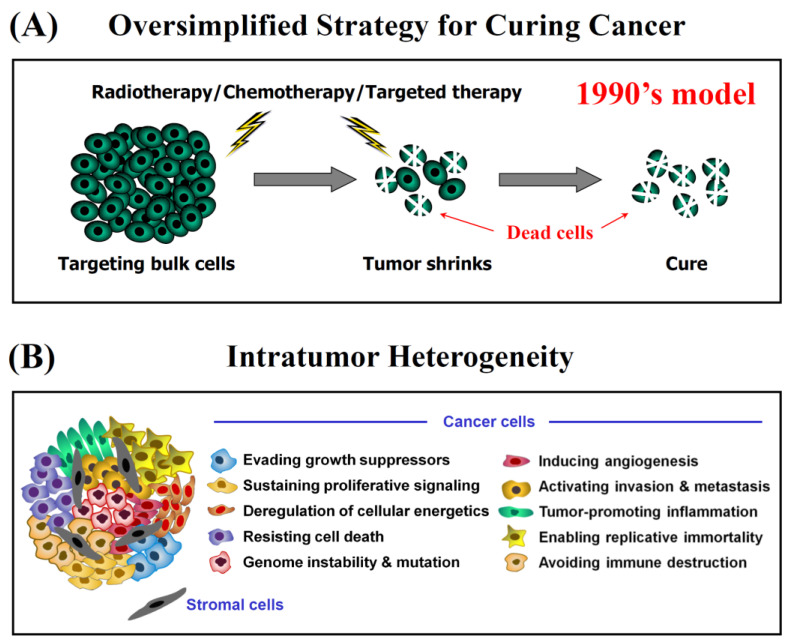
(**A**) Oversimplified (old) strategy for eradicating solid tumors through repeated doses of radiotherapy, chemotherapy, and other means (e.g., targeted therapies) when used singly or in various combinations; (**B**) Complex heterogeneity within an individual solid tumor (adapted from [91]).

The danger of oversimplifying cancer cell responses to therapeutic agents is illustrated by the cartoon in Figure 2. According to the model in panel A, initial anticancer treatment would kill the majority of cancer cells in an individual solid tumor, and subsequent treatment(s) with radiation and/or chemotherapeutic drugs (and more recently with targeted therapies) would be required for final eradication of the tumor.

Although this model now appears to be both overly simplistic and fundamentally flawed, it did provide the basis for the development of rapid (e.g., 48 h incubation with a test drug), high throughput multiwell plate colorimetric (e.g., tetrazolium-based; crystal violet-based) and fluorometric (resazurin-based, such as CellTiter-Glo) assays for the assessment of cancer cell death post-treatment. These and other widely used preclinical assays (including colony forming ability, immunoblotting, flow cytometry, tumor growth delay in live animals) are not designed to recapitulate the degree of cellular and molecular complexity and heterogeneity that exists within a single tumor (intratumor heterogeneity) (reviewed in, e.g., [4,91,92,93,94,95]; also see Figure 2B). Furthermore, continuous cell treatment with relatively high concentrations of chemotherapeutic drugs, which is required in order to induce a significant inhibitory effect (e.g., 50% “cytotoxicity”) in multiwell plate assays (see, e.g., [96,97,98,99,100,101,102,103]), often has little clinical relevance because many drugs are administered to a patient as a bolus [18,19]. Thus, as noted by Eastman, continuous incubation with a drug in laboratory studies “limits recovery whereas in the patient, the tumor is only exposed to the drug for a short period followed by time for recovery, a scenario that is too frequently overlooked in vitro” [18].

### 3.2. Publishing “Mansions of Straw” versus “House of Brick” Research Papers

Despite thousands of publications over the past few decades promising novel anticancer drugs and treatment strategies, the number of failures is staggering. The Nobel Prize Laureate William Kailen has outlined the reasons for this failure in a 2017 Nature World View article entitled “Publish Houses of Brick, not Mansions of Straw” [104] and in a more recent lecture entitled “Preclinical Cancer Target Validation: How Not to be Wrong” [105]. The presentation was part of the NIH Director’s Wednesday Afternoon Lecture Series, colloquially known as WALS, which is the highest-profile lecture program at the NIH. Kaelin discussed a number of important issues that have hampered progress in cancer therapy, including:

- Danger of cramming large amounts of data into a single manuscript, which often includes extensive data sets presented in the supplementary material, without taking the time to scrutinize what the results of each assay actually indicate. Kaelin uses the analogy of “building with straw” for such publications and states that the “real advances are built with bricks, not straw” [104].
- Danger of jumping to conclusions from ubiquitous use of “down” assays (e.g., decreased proliferation, decreased tumor growth, etc.) to clinical relevance. For example, as we see in numerous recent articles, if a test drug is shown to inhibit proliferation (e.g., in potentially highly misleading multiwell plate assays; see, e.g., [4,18]), down-regulate global levels of the protein under study in immunoblot analysis, and inhibit tumor growth in live (often immune deficient) animals, it is typically concluded that the test drug must have clinical relevance as a novel anticancer agent when administered with or without conventional therapies.
- Potential bias/misconception that can arise from the Kaplan–Meier survival curves and tumor growth delay assays in live animals when used to analyze prognostic/predictive molecular biomarkers of tumor response. Kaelin indicated that he sometimes calls this “the marriage of the gratuitous and the contrived” [105].

Kaelin’s 2018 presentation was mainly from the point of view of reductionist thinking. It was addressed to new students of cancer research, although the message is equally fascinating and thought provoking for experienced researchers. His remarks on Kaplan–Meier plots in the context of molecular biomarkers were particularly illuminating to us, given that as reviewers we do see such plots appearing in many papers to justify the clinical relevance of the protein/gene under study. Referring to such plots, he stated that “…you know full well… what the person has done is they searched as many databases as possible until they found one where the expression of their gene was linked to bad outcome and furthermore they’re trying to seduce you into thinking that not only is this correlative, this is causative. You better study my favorite gene; my favorite gene is important because it is associated with bad prognosis.”

The consequences of ignoring these cautionary notes became evident when the majority of candidate anticancer drugs failed during various phases of clinical trials and drug approval [2,106,107,108] (also see below).

### 3.3. Off-Target Effects of Anticancer Drugs Developed for Targeted Therapies

Papers published on the war on cancer over a decade ago expressed heightened excitement regarding major advancements in high content screens and rapid validation strategies to identify novel anticancer drugs capable of targeting specific cancer-associated proteins. This opened up the possibility of “targeted therapy” in which the drug would only modulate the pathway for which it was intended. One aspect of this scenario is called “synthetic lethality”, where perturbing two or more cellular targets concurrently would be expected to result in loss of cell viability (death), whereas perturbing only one of these targets would not [109]. Whether such target-specific approaches will have favorable clinical outcome for patients with solid tumors remains to be seen [2,109].

As mentioned above, the majority (~97%) of novel anticancer drugs that undergo clinical trials in oncology fail to advance to receive FDA approval. Using CRISPR/Cas9 mutagenesis, Lin et al. [106] investigated a set of cancer drugs and drug targets in various stages of clinical testing. Each drug was previously assumed to modulate the function of a specific protein (on-target effect). This study tested ten such drugs that were developed to target six proteins, five of which (HDAC6, MAPK14/p38α, PAK4, PBK, and PIM1) are known to represent cancer dependencies, and one protein (CASP3/caspase-3) that is the primary executioner in apoptosis. Some of these drugs were tested in a total of 30 clinical trials. Contrary to previous reports obtained predominantly with RNAi and small-molecule inhibitors, CRISPR/Cas9 experiments revealed that the proteins ostensibly targeted by all ten of the drugs that were tested were non-essential for cancer cell proliferation. Furthermore, the efficacy of each drug was unaffected by the loss of its putative target, indicating that the compounds were not functioning for the purpose they were developed (i.e., for “targeted therapy”) and had entered clinical trials. Based on these observations, Lin et al. [106] concluded that “stringent genetic validation of the mechanism of action of cancer drugs in the preclinical setting may decrease the number of therapies tested in human patients that fail to provide any clinical benefit.”

A different view was expressed when other small molecule inhibitors were shown to have off-target effects. Four inhibitors of the poly (ADP-ribose) polymerase (PARP) enzyme—olaparib, rucaparib, niraparib and talazoparib—are currently undergoing clinical trials as specific inhibitors of single-strand DNA break repair. Recently, Antolin et al. [110] demonstrated that each of these PARP inhibitors has an inherent capacity to inhibit off-target kinases, and concluded that their findings “emphasize the importance of comprehensive kinase profiling, using orthogonal technologies, of all candidate PARP inhibitors and in addition opens up potential new avenues for the rational design of dual PARP-kinase inhibitors with targeted polypharmacology.”

Although not alluded to in these articles, a novel cancer therapeutic strategy which does not take into account the responses outlined in Section 2, namely, the key roles played by apoptotic/dormant cancer cells in resistance and relapse, and overlooks intratumor heterogeneity which encompasses cancer immune surveillance (Figure 2B; also see below), is bound to generate undesirable clinical outcomes, at least for solid tumor therapy.

### 3.4. Cancer Immunoediting

The cancer immunoediting program, initially developed by Dunn et al. [111], represents the current state-of-the-art of understanding in cancer immunology [112,113,114,115]. It is extremely complex, involving three component phases (3E’s): elimination, equilibrium, and escape. Messerschmidt et al. [112] have provided a comprehensive review of these processes in a lay language which is easier to follow for non-immunologists like us.

In the elimination phase, innate and adaptive immune cells cooperate to destroy microcolonies of transformed cells. Sporadic transformed cells that manage to survive immune surveillance may then progress to the equilibrium phase where editing occurs. In the equilibrium process, cancer cells either remain in a quiescent state or proliferate, but then the immune cells fight back. The net outcome is that the tumor neither shrinks significantly in size nor grows.

The equilibrium phase, which is conceptually similar to clinical dormancy, is further subdivided into four different stages (for details, please consult [111]). It is also the longest phase of immunoediting, during which variants of dormant cancer cells are believed to be created which are kept under check by the immune system. It has been documented that circulating, disseminated tumour cells can survive in cancer patients for over 20 years without causing tumor outgrowth [116]. Rare cancer cells, however, can acquire genetic, epigenetic and metabolic changes that enable them to completely bypass immune surveillance and to progress to the escape phase of immunoediting. Such variants then grow to form a clinically detectable tumor with the potential of spreading and killing the patient. Thus, cancer cells that have achieved “immune escape” characterize most human cancers “that have reached a clinical stage that has attracted the attention of the patient and/or physician… Immune escape in a tumor is coincident with its observed malignant conversion” [112].

Several factors can contribute to cancer immune escape, including the “don’t eat me” signal CD47 (reviewed in [113]), which is upregulated in all human patient cancer cells that have been tested [117]. CD47 is an integrin-associated protein which binds to macrophage cell surfaces to inhibit phagocytosis [113]. In preclinical studies with animal models, targeting CD47 has been shown to remarkably inhibit the growth of human cancer xenografts [117,118,119,120]. Time will tell whether such an approach can significantly increase the survival of cancer patients when used singly or in combination with standard-of-care radio/chemotherapies.

*Take home message:* The interplay between the host immune system and pre-malignant/malignant cells is extremely complex, dynamic, and fundamental to the outcome of cancer therapy, and yet it is often overlooked in preclinical studies that are widely used to identify novel cancer therapeutics.

## 4. Dishonesty in Reporting Data Due to Pressure to Publish, and Not Reporting the Scientific Bases of Failed Clinical Outcomes for the Benefit of Drug Manufacturers

The concerns noted above mainly pertain to sloppiness in biomedical research, with too many published results turning out to be true only under narrow experimental conditions, if indeed they are reproducible at all.

It is reasonable to assume that the pressure to “publish or perish” can also lead to exaggerations about the significance or certainty of research findings and, even worse, publishing massaged or even falsified results, which often remains unnoticed, unless caught, in which case the paper is retracted. The consequence of such dishonesty is obvious, and extends beyond merely impacting the resume of the author(s). For example, as discussed in a Nature News article by Else Holly [121], two influential journals (Science and Nature) have recently retracted papers about DNA-repair processes after an investigation found that the papers contained falsified data. The papers, which were published in 2010 (Science) and 2013 (Nature), both reported important aspects of DNA double-strand break repair. This is terrible for the field, as it is for any field, in particular “because the investigator’s grants could have gone to more deserving researchers, says James Brown, a cancer researcher at the National University of Ireland Galway. Many scientists have used the Nature paper to build an understanding of DNA-repair processes mediated by a protein called KAT5 (also known as TIP60)…” [121].

Another culprit in biomedical cancer research is the lack of transparency of drug manufacturers and decision makers of cancer projects that lead to clinical trials. Maeda and Khatami discussed this “dark side” of cancer research in a Perspective article entitled “Analyses of Repeated Failures in Cancer Therapy for Solid Tumors: Poor Tumor-Selective Drug Delivery, Low Therapeutic Efficacy and Unsustainable Costs” that was published in Clinical and Translational Medicine in 2018 [2]. The authors presented a scientific analysis of the disturbing data on outcome failure rates of current cancer therapeutic approaches for solid tumors. Although in the last six decades minimal or partial success has been achieved with drugs such as Gleevec or a few other modalities, this limited success was only for the treatment of leukemia and non-solid or soft tissue tumors.

A common trend in the design of novel cancer therapies as presented by Maeda and Khatami [2] is summarized below.

Scientific analyses of data on the repeated failures of the majority of cancer projects involving novel chemotherapeutic drugs are rarely reported. These are highly publicized and well-funded projects that are claimed as ‘targeted’ therapies, ‘personalized’ or ‘precision’ medicine, or ‘immuno’ therapies. Such “novel” anticancer drugs and/or therapeutic strategies lead to clinical trials which end up with undesired outcomes for patients with solid tumors.

The decision makers of such expensive and out-of-focus undertakings either abandon data on failed clinical outcomes or disregard and downplay the serious consequences of drugs/treatment strategies that, at best, postpone a “patient’s death-sentence for a few months of remission” ([2] and references therein).

Once such expensively developed drugs (or “poisons” as Maeda and Khatami call them [2]) are found not to fulfill their promise, the clinical trial is suspended. Drug manufactures and decision makers often proceed by making some modifications to the same protocols, such as changes in dosage, route and frequency of drug administration, and/or use of combination drugs. Such strategies with modified “poisons” are again highly publicized as novel anticancer approaches “through control of media using the same empty promises to justify additional support for recruiting desperate patients in expensive schemes of clinical trials” ([2] and references therein).

In short, such an approach to identifying novel cancer drugs and therapeutic strategies has so far proven to be a major waste of research efforts, huge amounts of cancer research funds, and most importantly lives of desperate cancer patients [2].

## 5. Do Modern Cancer Therapies Prolong Survival of Patients with Solid Tumors?

In a recent comprehensive review on the impact of genome chaos on cancer progression and therapy resistance of solid tumors, Heng and Heng [122] wrote that “the main reason why cancer often wins battles in the war on cancer is its super evolvability. Ironically, this evolvability is greatly enhanced by our medical treatment strategies. In other words, in the name of killing monsters, we may be propagating them more, as treatment options designed for the maximal killing of cancer cells can result in the formation of more aggressive cancers.”

The mechanisms underlying the creation of increasingly aggressive “monsters” are multifactorial, extremely complex, but fairly well studied (as was briefly discussed in Section 2). These include the interplay between various manifestations of genome chaos (e.g., chromothripsis, polyploidy, multinucleation, micronucleation) [122], repeated loss of synchronization between circadian rhythms and cell cycle regulation [123,124], and reversal of cancer cells to an atavistic form of life, resulting in activation of resistance genes [54,123,124,125,126,127,128,129,130,131,132,133].

In the course of determining progress made in each of these responses since our last review that was published in 2020 [4], we encountered a blog on atavistic oncology which reported an observation that was surprising to us. The blog was posted in 2014, and presented a dispute between two accomplished oncologists, Frank Arguello (Cd Juárez, Chih, Mexico; former Senior Scientist, Division of Cancer Treatment & Diagnosis, National Cancer Institute, NIH, Frederick, MD, USA) and David Gorski (Wayne State University School of Medicine and Barbara Ann Karmanos Cancer Institute, Detroit, MI, USA), on the atavistic model of cancer [134]. (Since 2014 there has been significant progress in this field of research; see, e.g., [54,123,124,125,126,127,128,129,130,131,132,133]). What we found particularly fascinating in this blog concerns the life expectancy of patients with a given solid tumor nearly a century ago as compared to that in the modern era.

In a letter dated August 2014 which is now in the public domain [135], Arguello wrote “I have been studying the survival time of people with cancer who were never treated with our modern approaches of chemotherapy and radiation, not even surgery… I collected papers from the 1800s and the first decades of the 1900s discussing survival time of untreated cancer patients. In the case of esophageal cancer, I found an article which reports the median survival time of 74 untreated patients with esophageal cancer seen at Middlesex Hospital, London, England, between 1883 and 1922. It reports an overall median survival time of 14.7 months… [136]. These are patients who did not receive any form of surgical treatment (medical treatments obviously did not exist at that time other than opium)… In a recent, 2014, study in the Netherlands involving 127 patients with inoperable or irresectable esophageal cancers, patients were divided into two groups—one group received chemotherapy and radiation and the other group radiation alone. The median survival time was 14 and 9 months, respectively [137]. In theory, we should be doing far better today in 2014 than 100 years ago, because aside from chemotherapy and radiation, we have potent antibiotics, blood transfusions, parenteral feeding, gastrostomies, stents, intensive care units, etc., which did not exist in the late 1800s and early 1900s. However, this does not appear to be the case.”

Gorski argued with this evaluation by pointing out several reasons why such a comparison cannot be meaningfully made [135]. With regard to comparing esophageal cancer outcomes in 2014 with 1924 and earlier, particularly cancer from 2014 that is inoperable, he stated that what constitutes “inoperable” today is very different from what constituted “inoperable” 100 years ago. He noted that “It’s a huge difference. Modern surgical technique allows us to remove tumors that no surgeon could have removed 100 years ago without killing the patient.”

*Take home message:* While both of these oncologists have valid points, we find the merely modest difference in life expectancy of esophageal cancer patients over the span of a century not entirely surprising, in part because now we know that surgery can trigger tumor growth and metastases (see below). Furthermore, treatment options for solid tumors that “are designed for the maximal killing of cancer cells can result in the formation of more aggressive cancers” [122]. Indeed, a very disturbing picture emerges when considering the fact that all of the aforementioned therapy-induced responses that underlie recurrence (e.g., genome chaos, reversal of dormancy, anastasis, cell fusion, reverse evolution) can occur in different subsets of cancer cells within a given solid tumor. Collectively, these discoveries make one wonder if modern cancer therapies cause more harm than benefit.

## 6. Impact of Surgery on Tumor Progression

Once a patient is diagnosed with an apparently localized solid tumor, an immediate surgery is crucial to decrease the risk for accelerated growth of micrometastatic disease and increased formation of new metastatic foci. Despite this unquestionable benefit, the unwanted side effects of surgery for cancer patients is becoming increasingly appreciated [138,139,140,141,142]. In 2017 Tohme et al. [140], for example, reviewed the often fragmentary clinical and experimental evidence supporting the potential role of surgery itself on tumor recurrence. An obvious downside of surgical removal of solid tumors is the increased risk of shedding of cancer cells into the circulation. This shedding “suppresses anti-tumor immunity allowing circulating cells to survive, upregulates adhesion molecules in target organs, recruits immune cells capable of entrapping tumor cells and induces changes in the target tissue and in the cancer cells themselves to enhance migration and invasion to establish at the target site” [140]. In addition, these authors discussed the potential influences of preoperative factors (e.g., anesthesia, transfusions, hypothermia), postoperative complications and surgical trauma-associated local and systemic inflammatory responses on early cancer recurrence [140].

After removal of solid tumors, might post-surgery wound healing processes also contribute to disease recurrence? In 2018 Weinberg’s group reported a series of studies with an experimental mouse model system that were designed to address this question [141]. For this purpose, outgrowth of D2A1-GFP (mouse breast cancer) cells was determined under the following three conditions: (i) cells injected directly into the wound-healing microenvironment, which was created by subcutaneously implanting sterile polyvinyl acetate sponges one week prior to cell injection; (ii) cells injected orthotopically into the mammary fat pads (MFPs) of wounded animals; and (iii) cells injected orthotopically into MFPs of unwounded (control) animals. Tumor outgrowth was observed only in wounded animals, and outgrowth was more robust in the wound-healing site than in the MFPs. Furthermore, pre- and post-operative treatment of animals with meloxicam, a nonsteroidal anti-inflammatory drug, attenuated the impact of the surgical wound healing process on tumor outgrowth. The clinical implications of these observations remain to be elucidated.

There is another potential dark side of surgery that is merely based on observational data. As discussed by Goldstein and Mascitelli [138], occult microscopic cancers in different organs (e.g., prostate, breast) are exceedingly common in the general population. Such cancers are believed to be held in a quiescent state by a balance between cell proliferation and cell death and also an intact host immune surveillance, and thus never cause symptoms and complications. It is possible that surgical insult for purposes not related to cancer might trigger these occult cancers to become malignant.

## 7. Where Do We Go from Here in the War on Cancer?

After discussing the current dilemma of cancer research, stemming from the logical reductionist thinking and data generating approach, Weinberg concluded his 2014 article [3] by writing “How will all this play out? I wouldn’t pretend to know. It’s a job, as one says on these occasions, for the next generation. Passing the buck like this is an enormously liberating experience, and so I’ll keep on doing it!”

Eight years later, what lessons can we teach the next generation of students? Where do we go from here in the war on cancer? Will it ever be possible to prevent disease recurrence following treatment of patients with solid tumors?

Like Weinberg, we wouldn’t pretend to know, but suggest the following steps as a good starting point, based on emerging trends including those highlighted above (Section 2):

- Application of single cell assays, preferably time lapse microscopy, to detect different subgroups of cancer cells within a tumor and to follow their fates after therapeutic exposure [4,8,143,144].
- Employing cell-based assays that distinguish dead cancer cells and dying cancer cells (e.g., exhibiting features of apoptosis) that have the potential to recover from the brink of death [8,144].
- Using anticancer drug exposure in preclinical studies (e.g., cell-based assays) under conditions (drug concentrations, treatment and post-treatment incubation times) that are consistent with how drugs are administered to patients (see, e.g., [18,19]).
- Regarding cancer as a network phenomenon (e.g., [145,146,147,148]). To this end, as recently pointed out by Erenpreisa and Giuliani [147], “The reductionist, one gene/one protein method that has served us well until now, and that still dominates in biomedicine, requires complementation with a more systemic/holistic approach, to address the huge problem of cross-talk between more than 20,000 protein-coding genes, about 100,000 protein types, and the multiple layers of biological organization.”
- Abandoning the use of immune deficient/compromised animal models in anticancer-related studies, or at least being aware of their limitations (see below).

The preclinical validation of a novel anticancer therapeutic with respect to entering the FDA approval process typically involves the generation of both in vitro/tissue culture and in vivo data, the latter commonly using mouse models of human cancer. In addition to the limitations discussed in Section 2, there is also an important potential shortcoming of the standard in vivo models which typically involve either human cancer cell lines (xenografts) or human tumor tissue (patient-derived xenograft or PDX) engrafted into immune-deficient mouse strains to circumvent rejection of the grafts by the host immune system. In the past decade it has become evident that these standard mouse models of human cancer may in fact be poorly informative for generating such preclinical data because the host-/patient-generated anti-tumor immune response is being increasingly recognized as a potential contributor to the tumor response to both radiation therapy (e.g., [149,150,151,152,153]) and some cytotoxic chemotherapy drugs (e.g., [154,155,156,157,158,159,160,161,162,163,164,165]).

We are now at the dawn of a new age of opportunity where immune-competent mouse models have been developed using a variety of approaches to partially reconstitute key elements of the host immune system (e.g., [149,166,167,168,169]). Indeed, humanized mice and mice whose immune system has been partially recreated with human immune elements are now available commercially through vendors such as the Jackson laboratory [170], albeit at a considerable cost. These models would also allow researchers to address a number of unresolved questions such as the impact of a functional immune response and its stimulation by immune checkpoint inhibitors on potentially detrimental cancer cells that have undergone therapy-induced dormancy through senescence or PGCC formation, as was discussed in Section 2.3.

As pointed out by Maeda and Khatami [2], “Effective cancer immunotherapy requires systematic understanding of the mechanisms that contribute to the ability of tumor cells to escape and bypass the immune surveillance by induction of decoy receptors, enhanced immune tolerance and loss of mitochondrial function (mitophagy), altered anabolic (growth-promoting) and catabolic (necrosis or growth-arresting) recycling proteins/lipids pathways (autophagy) in tissues. These interdependent complex pathways were defined to be provided through the two biologically opposing arms of Yin (tumoricidal, apoptosis, growth arrest) and Yang (tumorigenic, wound healing or growth promoting) pathways of acute inflammation or effective immunity.” The availability of the aforementioned “humanized” animal models will hopefully lead to designing strategies to significantly tip the Yin-Yang balance in order to profoundly improve the outcome of cancer therapy.

## 8. Conclusions

In the Nature Editorial article on the war on cancer [1] mentioned above (Section 1), the authors concluded that “The combination of technological advances with continued collaboration between basic and clinical researchers could sustain the momentum generated by the act into the next 50 years—and take the field ever further from the days when surgery and radiotherapy were the only treatments.”

The aim of our current article was not to propose a therapeutic strategy per se, but rather to highlight sufficient discoveries to enable the reader to elaborate or debate on the main conclusion that we have reached. Namely, the majority (thousands) of cancer therapy-related articles that employ conventional preclinical anticancer assays (e.g., multiwell plate viability, colony formation, apoptosis, tumor growth kinetics in live animals) often report dangerously misleading and highly biased “science fiction” rather than science of any clinical relevance, since they disregard cellular complexity and heterogeneity within a single tumor and the impact of the immune system on the outcome of anticancer treatment.

There is an urgent need for designing preclinical anticancer assays (both cell-based and animal models) and treatment strategies that recapitulate the degree of complexity that exists within an individual solid tumor. Until then, at least in the near future, perhaps efforts of cancer researchers should be primarily directed towards prevention, rather than employing the same misleading preclinical assays and wishy-washy interpretations (to quote Kaelin [105]) with “novel” anticancer drugs and catchy names for treatment strategies (e.g., “synthetic lethality”) to expect different outcomes in the next 50 years.

Focusing on prevention was proposed by Coleman [171] and others (e.g., [172]) nearly a decade ago. In 2013 Coleman wrote “The language of war, victory and defeat is misplaced and outdated… we should drop the lazy, simplistic jargon and the distorted priorities of ‘war’, and focus on prevention to make long-term progress against cancer” [171].

Accumulating evidence suggests that clues for identifying means of preventing the development of the malignant disease might be obtained by studying long-lived animals, most notably the subterranean rodent species naked mole rat (NMR) (reviewed in, e.g., [173,174,175]). These animals live for decades under harsh, hypoxic environments and yet remain cancer free. This is particularly remarkable because hypoxia is a robust trigger for the development of PGCCs, the “evil roots” of human cancer [57]. Some authors have proposed that uncovering the molecular mechanisms of cancer resistance in NMR and other long-lived animals may also lead to the development of novel cancer therapies (e.g., [173,174]).

## Appendix A

DAPI (4′:6-diamidino-2-phenylindole) images showing the development of polyploid, multinucleated, and micronucleated giant cells in the SUM159 breast carcinoma cell line after exposure to ionizing radiation and incubation for 3 days. Scale bars, 20 µm. Reproduced from Mirzayans and Murray [26].

## Appendix B

Phase-contrast photomicrographs showing premature senescence in MDD2, a mutant p53-expressing derivative of the MCF7 breast carcinoma cell line. Cultures were exposed to ionizing radiation (8 Gy) or sham-irradiated (control), incubated for seven days, and evaluated for flattened and enlarged cellular morphology and positive (blue) staining in the senescence-associated β-galactosidase assay. Reproduced from Mirzayans et al. [50].
